# Mitigating the attachment of *Salmonella* Infantis on isolated poultry skin with cetylpyridinium chloride

**DOI:** 10.1371/journal.pone.0293549

**Published:** 2023-12-21

**Authors:** Dana K. Dittoe, Elena G. Olson, Lindsey A. Wythe, Zachary G. Lawless, Dale R. Thompson, Lindsey M. Perry, Steven C. Ricke

**Affiliations:** 1 Department of Animal Science, University of Wyoming, Laramie, Wyoming, United States of America; 2 Department of Animal and Dairy Science, University of Wisconsin-Madison, Madison, Wisconsin, United States of America; 3 Department of Computer Science and Computer Engineering, University of Arkansas, Fayetteville, Arkansas, United States of America; 4 Safe Foods Corporation, Little Rock, Arkansas, United States of America; University of Illinois Urbana-Champaign, UNITED STATES

## Abstract

To provide the poultry industry with effective mitigation strategies, the effects of cetylpyridinium chloride (CPC) on the reduction of *Salmonella* Infantis, *hilA* expression, and chicken skin microbiota were evaluated. Chicken breast skins (4×4 cm; N = 100, n = 10, k = 5) were inoculated with *Salmonella* (Typhimurium or Infantis) at 4°C (30min) to obtain 10^8^ CFU/g attachment. Skins were shaken (30s), with remaining bacteria being considered firmly attached. Treatments were applied as 30s dips in 50 mL: no inocula-no-treatment control (NINTC), no treatment control (NTC), tap water (TW), TW+600 ppm PAA (PAA), or TW+0.5% CPC (CPC). Excess fluid was shaken off (30s). Samples were homogenized in nBPW (1 min). Samples were discarded. *Salmonella* was enumerated and Log_10_ transformed. Reverse transcriptase-qPCR (rt-qPCR) was performed targeting *hilA* gene and normalized using the 2^-ΔΔCt^ method. Data were analyzed using one-way ANOVA in RStudio with means separated by Tukey’s HSD (P≤0.05). Genomic DNA of rinsates was extracted, 16S rRNA gene (V4) was sequenced (MiSeq), and data analyzed in QIIME2 (P≤0.05 and Q≤0.05). CPC and PAA affected *Salmonella* levels differently with CPC being effective against *S*. Infantis compared to TW (P<0.05). Treatment with CPC on *S*. Infantis-infected skin altered the *hilA* expression compared to TW (P<0.05). When inoculated with *S*. Typhimurium, there was no difference between the microbiota diversity of skins treated with PAA and CPC; however, when inoculated with *S*. Infantis, there was a difference in the Shannon’s Entropy and Jaccard Dissimilarity between the two treatments (P<0.05). Using ANCOM at the genus level, *Brochothrix* was significant (W = 118) among skin inoculated with *S*. Typhimurium. Among *S*. Infantis inoculated, *Yersiniaceae*, *Enterobacterales*, *Lachnospiraceae CHKCI001*, *Clostridia vadinBB60 group*, *Leuconostoc*, *Campylobacter*, and bacteria were significant (40<W>8). CPC and PAA-treated skins had lowest relative abundance of the genera. In conclusion, CPC mitigated *Salmonella* Infantis, altered *hilA* expression, and influenced the chicken skin microbiota.

## Introduction

The occurrence of microbial adhesion to poultry carcass skin is well known and documented with a diversity of bacterial cells capable of attaching to poultry skin under analogous conditions [1–6]. Aerobic bacteria and *Enterobacteriaceae* are firmly attached to chicken carcasses before arriving at the processing facilities [7]. Although bacterial counts per gram of chicken skin decrease with each processing step, firmly attached bacteria are not eliminated by rinsing methods [7, 8]. Numerous data show that bacterial cells attached or entrapped on poultry skin are more impenetrable to various antimicrobials [1–7]. The durability of *Salmonella* on fresh manufactured poultry skin and the complications confronted in eradicating the foodborne pathogens from processed meats may be due to the complexity of eliminating these attached cells by generally used washing and rinsing strategies [7]. In addition, it has been noted that outside factors such as chilling poultry carcasses via aeration or in water, a method typically exercised in the United States, may aid the attachment of *Salmonella* to chicken skins [9, 10].

Post-chill application of antimicrobials has been used as one of the primary methods for reduction of pathogens [10]. Over ten antimicrobials are approved in poultry manufacturing during the post-chill step, with peracetic acid (PAA) and cetylpyridinium chloride (CPC) being considered the leading agents [10]. The use of PAA as an antimicrobial agent has been used extensively in the poultry industry due to its high oxidizing ability. Bertram et al. [11] noted that PAA achieved better antimicrobial results on chicken skin compared to chicken meat, most likely due to the unique composition of the skin. The quaternary ammonium sanitizer CPC has been frequently examined in numerous studies and has been described as a successful antimicrobial for poultry meats and surfaces at a maximum concentration of 0.8% [12]. Ma et al. [13] demonstrated that CPC was able to disrupt the bacterial respiration cycle and affect the morphology of *Salmonella*. Furthermore, Yegin et al. [12] noted that reducing *Salmonella* on chicken skin using CPC could be partly due to the electrostatic connection of negatively charged surfactant (CPC) with the positively charged outer membrane of Gram-negative bacteria. Nevertheless, scientific research does not examine antimicrobial results of CPC versus foodborne pathogens such as *Salmonella* on poultry skin [12].

The *hilA* gene of *Salmonella* spp., the central pathogenesis regulator across pathogenetic *Salmonella* spp. and virulence indicator [14], has been utilized as a marker for overall virulence and stress responses by pathogenic *Salmonella* spp. in the presence of environmental stressors such as pH and desiccation and its expression is known to vary considerably among serovars [15]. As such, using *hilA* to understand the impact of processing antimicrobials is ever important, especially with virulence being impacted by acid stress [15] which could be imparted by the use of PAA, an organic acid with a pH of 3 to 5 at concentrations ranging from 50 to 1,000 ppm [10, 16].

In 2023, Rasamsetti and Shariat [17] reported that *Salmonella enterica* serovars Typhimurium, Infantis, and Kentucky were the most commonly isolated and identified serovars among poultry carcass rinsates collected at commercial broiler processing facilities. Additionally, through CRISPR-seroseq, Rasamsetti and Shariat [17] demonstrated the re-immersion of *S*. Infantis after carcass cut up on broiler drumsticks before antimicrobial interventions being applied. *S*. Infantis is becoming a significant public health and food safety concern. It has been reported that the *S*. Infantis serovars isolated from broilers carry a large conjugative mega-plasmid pESI (plasmid for emerging *S*. Infantis), which can provide bacterial cells with two to five drug resistance genes [18, 19]. However, due to the decrease in the number of *Salmonella*-positive samples among whole bird carcass rinse samples collected post-chill due to antimicrobial use in the processing facilities, it is difficult to investigate the serovar-specific response to antimicrobials [17]. Understanding the impact of these processing interventions on emerging *Salmonella* serovars due to the difference in virulence is increasingly important.

Beyond evaluating the effect of antimicrobials on the virulence of *Salmonella* spp., determining the impact of these processing interventions on the microbiota of poultry skin is equally as important. Dittoe et al. [20] demonstrated the reduced shelf-life of poultry parts due to microbiota shifts in response to PAA. Previously, we had demonstrated that CPC was potentially effective against *S*. Infantis on the skin surface of bone-in chicken thighs and altered the surface microbiota [21]. In the current study, we chose to evaluate the impact of CPC on the more firmly attached *S*. Infantis and skin microbiota after the removal of the skin from chicken breasts. This allowed us to directly assess antimicrobial impacts on, the skin microbiota and *Salmonella* virulence expression. Therefore, the current study assessed the antimicrobial effects of CPC (0.5%) on chicken skin against firmly attached *Salmonella* Infantis as compared to PAA (600 ppm). More specifically, when applied directly to isolated chicken skins, we evaluated the response of *Salmonella* spp., *hilA* expression, and microbiota diversity and composition to CPC. As *S*. Typhimurium is one of the most common *Salmonella* spp. isolated from poultry meat [17, 22], it is commonly used to demonstrate the efficacy of poultry processing interventions such as PAA. *Salmonella* Typhimurium has also been examined previously for responses to antimicrobial treatment when adhered to chicken skin [23]. Thus, *Salmonella* Typhimurium was used as a control measure in the study against *Salmonella* Infantis both on the level of *hilA* expression and microbiota compositional profiles.

## Materials and methods

### Chicken procurement and preparation

**Fig 1:**
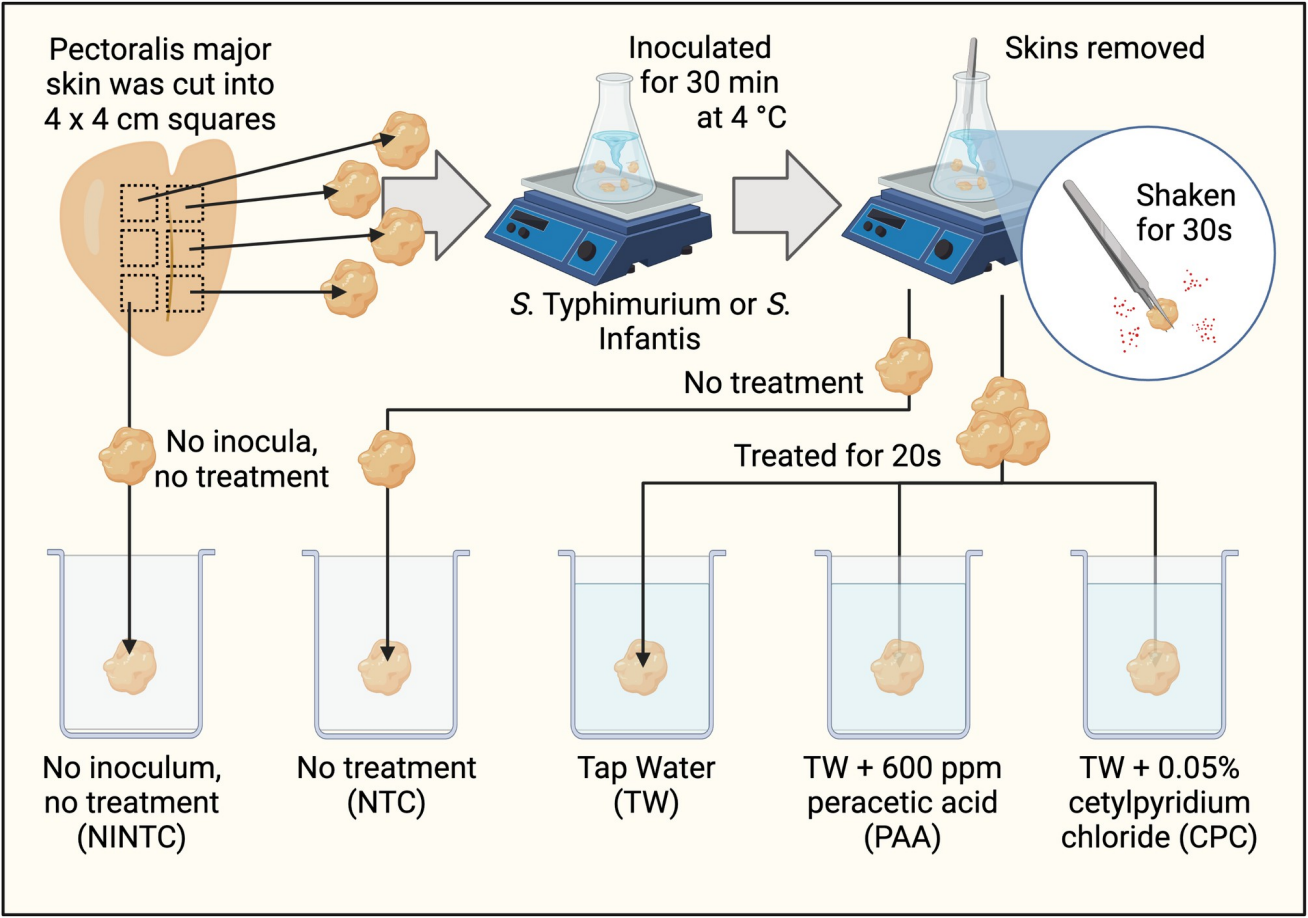
Schematic of experimental procedures. The skin of the Pectoralis major was cut using sterile forceps into 6 separate 4 × 4 cm squares that were inoculated with either *Salmonella* Typhimurium or Infantis and allowed to attach for 30 min at 4°C. Skins were removed and shaken for 30s to remove non-firmly attached *Salmonella*. Skins were treated for 20s in respective treatments, stomached for 1 min at 200 rpm and *Salmonella* was enumerated, the *hilA* expression quantified, and the V4 region of the 16S rRNA gene was sequenced. Created with BioRender.com.

The following experiment was conducted two independent times with either *S*. Typhimurium or *S*. Infantis being inoculated. For each serovar, a total of ten chicken carcasses were obtained post-chill (immersion) from the University of Arkansas commercial pilot processing plant from one flock (Fayetteville, Arkansas; n = 10). As no live animals were utilized in the current study and poultry carcasses were collected during routine processes, the current study was exempt from the Institutional Animal Care and Use Committee. Carcasses were transported in sterile whirl pack bags on ice to the Center for Food Safety at the University of Arkansas, Fayetteville, Arkansas. Once in the laboratory, the skin of the pectoralis major (breast) was removed aseptically and cut into 4 × 4 cm squares using sterilized scalpels on a sterile commercial cutting board. In total, six squares were cut from the skin and five of those 4 × 4 cm squares were randomly assigned to the five treatments in the current study: no inocula, no treatment control (NINTC), no treatment control (NTC), tap water (TW), TW + 0.5% CPC, and TW + 600 ppm PAA (k = 5; **Fig 1**). This methodology was repeated for all ten carcasses per each inocula, *S*. Infantis and Typhimurium (N = 100, n = 10, k = 5, trial = 2). All instrumentation was cleaned and sanitized between biological replicates.

### Inocula preparation

Inocula were prepared according to Wythe et al. [21]. Before the onset of the current study, frozen stocks of *Salmonella* Typhimurium (ATCC 14028) and Infantis (CDC H3536) that were selected to be resistant to 64 μg/mL of nalidixic acid (NA) were streaked for isolation on Tryptic Soy Agar (TSA; EMD Millipore, Burlington, MA) and incubated for 24 h aerobically at 37°C. Subsequently, one isolated colony from the incubated plates was streaked onto fresh TSA with the addition of 64 μg/mL of NA (TSA + NA) and incubated under the previously mentioned conditions. Simultaneously, an isolated colony was streaked onto XLD (HiMedia, Kennet Square, PA) plus 64 μg/mL of NA (XLD + NA) for confirmation and incubated aerobically at 37°C for 24 h. Ten individual isolated colonies were subsequently transferred to ten separate conical tubes containing 40 mL of fresh Tryptic Soy Broth with 64 μg/mL of NA (TSB + NA) and incubated aerobically at 37°C in a shaking incubator at 200 rpm for 14 h. Directly following the overnight (14 h) incubation, the cultures (10^9^ CFU/mL) were centrifuged at 18,000 g for 5 min, decanted, and washed twice in 1 × Phosphate Buffered Saline (PBS; 8 g of NaCl, 0.2 g of KCl, 1.44 g of Na_2_HPO_4_, and 0.24 g of KH_2_PO_4_ per 1 L, with the pH adjusted to 7.4 with HCl). After the final wash, the pellet was resuspended in 40 mL of PBS and diluted (1:10) in an Erlenmeyer flask (1 L) containing 360 mL of PBS. The resulting inocula (400 mL) were determined to contain 10^8^ CFU/mL of either *S*. Typhimurium or Infantis via spread plating on XLD. The methodology was the same across both serovars.

### Inoculation

Per biological replicate, 4–4×4 cm skin samples were placed into Erlenmeyer flasks (1 L) containing either 400 mL of resuspended *S*. Typhimurium or Infantis and placed into a 4°C cold room on stir plates (200 rpm) (**Fig 1**). The fifth skin sample remained uninoculated as the no inocula, no treatment control (NINTC). Skin samples were incubated for 30 min at 4°C to allow for attachment. Using sterile forceps, inoculated skins were shaken for 30 s to remove excess fluid and placed into sterile collection bags. Any bacteria remaining were considered firmly attached as defined by Lillard [7].

### Treatment application

Stocks of the two antimicrobial treatments, CPC and PAA, were created at the onset of the experiment by combining 15 L of tap water with the appropriate amounts of cetylpyridinium chloride (CPC, Cecure^®^, Safe Foods Corporation, North Little Rock, AR, USA) or peracetic acid (PAA, Promoat^™^, Safe Foods Corporation, North Little Rock, AR, USA) to obtain 0.5% CPC and 600 ppm PAA [21]. The treatments CPC and PAA were aliquoted (50 mL) to sterile collection bags. After inoculation, the following treatments were applied as a short-duration antimicrobial dip following methods described by Wythe et al. [21]: a no-treatment control (NTC), a 20 s application of tap water alone (TW), TW with the addition of 0.5% CPC (CPC); and TW with the addition of TW + 600 ppm PAA (PAA). A no inocula, no treatment control (NINTC) was maintained throughout the study. All treatments were administered at room temperature. Skins were shaken using sterile forceps for 30 s to remove excess fluid (**Fig 1**) [12]. The remaining bacteria were considered firmly attached. The level of firmly attached *Salmonella* was determined to be between 7.7 and 8.2 Log_10_ CFU/g as demonstrated on skins designated at the NTC.

### Sampling methodology

Following the treatment, the samples were transferred to new sterile collection bags. Immediately after, the skins were rinsed with 20 mL of sterile neutralizing Buffered Peptone Water (nBPW; pH 7.7; 20.0 g of buffered peptone, 7 g of refined soy lecithin or equivalent, 1.0 g of sodium thiosulfate, 12.5 g of sodium bicarbonate, per 1 L of DI water) [24]. The Whirl-Pak bags containing the 20 mL of nBPW and samples were stomached for 1 min at 200 rpm, the skins were discarded, and the resulting rinsates were collected for downstream analysis (*Salmonella* enumeration, reverse transcriptase (rt)-qPCR, and microbiota sequencing).

#### *Salmonella* enumeration

Approximately 20 μL of aliquoted rinsates were serially diluted to 10^−6^ (1:10 dilution factor) in 180 μL of 1× PBS in a 96-flat bottom plate. After diluting the samples, a 10 μL aliquot of each dilution was spot-plated onto XLD + NA (64 μg/mL) agar in duplicate and allowed to dry completely before inverting [25]. The plates were incubated aerobically for 24 h at 37°C. Only the plated dilutions with between 6 and 60 CFU counts were enumerated and recorded [25]. Therefore, the limit of quantification in the current experiment was 6 × 10^2^ Log_10_ CFU/mL.

### Reverse transcriptase PCR

The RNA of the rinsates was extracted using the Qiagen RNeasy Mini Kit (Qiagen, Valencia, CA, USA) using the protocol for animal cells. The RNA concentration was measured using a NanoDrop ND-1000 (Thermo Scientific, Wilmington, DE). The RNA was diluted to 10 ng/μL in RNAse-free water. Primer/probe assay targeting the *hilA* (experimental gene) gene was developed utilizing IDT PrimerQuest Design Tool (IDT, Newark, NJ). The *hilA* (cy5) and 16S (FAM; internal control gene) [26] genes were ordered from IDT as PrimeTime^™^ primer/probe assays (probe conc. 12.5 nm, forward conc. 25.0 nm, reverse conc. 25 nm; **Table 1**). Primers were reconstituted in 1× Tris-EDTA (TE, pH 8.0; Promega, Madison, WI) as a 40× concentration. Reverse transcriptase qPCR was performed on a CFX-96 (Bio-Rad, Hercules, CA) using the one-step rt-qPCR kit according to the standard protocol. The reaction volume was 10 μL with 5 μL of iTaq universal probes reaction mix (2×), 0.25 μL iScript advanced reverse transcriptase, 0.25 μL *hilA* primer/probe assay, 0.25 μL 16S primer/probe assay, 2 μL of RNA template, and 2.25 μL of RNAse free water. Standard curves of pooled samples (5 μL/sample) were maintained on each plate to determine PCR efficiency. A standard curve was developed from a 1:2 dilution series (1:2, 1:4, 1:8, 1:32, 1:64) of the pooled samples. The PCR efficiency was maintained between 90 and 110%, and the standard curves of the pooled samples were targeted at -3.3 with an optimal R^2^ greater than 0.99 for both primer/probe assays. Cycle thresholds were obtained from the Bio-Rad CFX-96, and fold expression change was presented using the Livak method [27], with *hilA* expression of total 16S being calculated using the following equation with the no treatment control (NTC) represented as the control conditions:

$$\text{Fold}\;\text{Expression}\;\text{Change}=2^{\left(-\left(hilA_{Treatment}-16S_{Treatment}\right)-\left(\;\text{hil}A_{meanNTC}-16S_{meanNTC\;}\right)\right)}$$

**Table 1:** The qPCR primer/probes assays that were used in the current study.

Target	Fluorophore	Sequence		Reference
hilA	Cy5	Forward	TTTGCTCAGATGTATTCCCGG	This Study
		Reverse	CTGTTTGGCGACATGTTAACG	
		Probe	TCTGCTTTGTGTCCCAGCGAAGT (IBRQ^1^)	
16S	FAM	Forward	ATGGCTGTCGTCAGCT	Harms et al., 2003
		Reverse	ACGGGCGGTGTGTAC	
		Probe	CAACGAGCGCAACCC (ZEN/IBFQ^2^)	

^1^IBRQ: Iowa Black RQ

^2^ZEN/IBFQ: ZEN/Iowa Black^™^ FQ

### DNA extraction

According to methods described by Wythe et al. [21], samples from all treatments and inocula were collected for microbiota analyses and stored in 1.5 mL microcentrifuge tubes at -20°C until they were processed. Samples were centrifuged (Heraeus Pico 21, Thermo Fisher Scientific, Langenselbold, Germany) for 10 min at 5,000 x g to pellet the rinsates. Pellets were homogenized in 180 μL of ATL buffer (Qiagen, Valencia, CA) and following the Gram-Negative bacteria protocol of the QIAamp DNeasy Blood and Tissue Kit (Qiagen, Valencia, CA), the genomic DNA was extracted. Final elution was performed in buffer AE (Qiagen, Valencia, CA). The DNA concentration and purity was determined using a NanoDrop ND-1000 (Thermo Scientific, Wilmington, DE). If the concentration of DNA exceeded 15 ng/μL, the samples were diluted to 10 ng/μL of DNA in buffer AE (Qiagen, Valencia, CA) and stored at −20°C until PCR amplification occurred.

### Library preparation and microbiota sequencing

Based on Kozich et al. [28], 16S rRNA gene sequencing libraries targeting the V4 region were prepared using dual-indexed primers. Amplification of the V4 region of the 16S rRNA gene was done using a high-fidelity polymerase, AccuPrime *Pfx* DNA polymerase (Invitrogen, Carlsbad, CA, United States), on an Eppendorf Mastercycler X50a (Eppendorf, Hamburg, Germany) using the following protocol: initial denaturation at 95°C for 5 min and 35 cycles of 95°C for 15 s (denature), 55°C for 30 s (anneal), and 68°C for 1 min. The ramp speed was uniformly set at 1°C/s. Using gel electrophoresis, the amplification of the targeted region of all samples and the non-amplification of the no template control were confirmed. Confirmed amplified amplicons (18 μL) were normalized using a SequelPrep^™^ Normalization Plate Kit (Invitrogen, Waltham, MA, United States) to achieve an equimolar concentration and equal volume of the samples (∼0.8 ng/μl). Normalized samples were pooled (5 μL/sample) to construct the final library. The final library was quantified using a Qubit fluorometer using the dsDNA broad-range assay (Thermo Fisher Scientific, Waltham, MA, United States) and quantitative PCR using the KAPA qPCR library quantification kit (Kapa Biosystems, Inc., Wilmington, MA, United States). An Agilent 2100 Bioanalyzer System (Agilent, Santa Clara, CA, United States) was used to confirm amplicon size.

Separately, the library and PhiX Control V3 (Illumina, San Diego, CA) were denatured with 0.2 N NaOH diluted to 20 pM with HT1 buffer. Library and PhiX were further diluted to 6 pM in HT1 buffer (Illumina, San Diego, CA) and combined (10% PhiX). Diluted and combined library (90% wt/vol) and PhiX control (10% wt/vol) were loaded into a Miseq V2 cartridge and sequenced on an Illumina Miseq as per standard Illumina practices (Illumina, San Diego, CA). The resulting sequences (fastq files) were downloaded from BaseSpace (Illumina, San Diego, CA) and uploaded to NCBI Sequence Read Archive (BioProject Accession: PRJNA899921; ID: 899921) and GitHub (https://github.com/RickeLab-UW/CPC-on-Salmonella-Infantis).

### Statistical & bioinformatic analyses

#### Statistical analyses

Each skin sample was randomly assigned to treatment prior to analyses. The CFU of *Salmonella* were Log_10_ transformed, reported on a Log_10_ CFU of *Salmonella* per g of skin basis [29], and imported into R Studio (R Studio version 1.4.1103; R version 4.0.3). Using the Livak (2^-ΔΔCt^) method with tap water (TW), PAA, and CPC as the treated experimental conditions, the no treatment control (NTC) as the control experimental condition, *hilA* as the experimental gene, and 16S as the control gene, fold expression data were Log_10_ transformed and analyzed in R studio. The data were analyzed using one-way ANOVA with pairwise differences assessed statistically by using Tukey’s protected HSD at a 0.05 level of significance.

#### Bioinformatic analyses

The sequenced data from both trials (N = 84, n = 5–10) were downloaded from Illumina BaseSpace, de-multiplexed, and locally uploaded into QIIME2-2023.2 [30]. Sequences were filtered for quality and trimmed in DADA2 via q2-dada2, with chimeras filtered by consensus and quality [31]. The phylogenetic trees were generated with fragment-insertion sepp [32–35]. Taxonomic alignment was performed using classify-sklearn against the Silva 128 SEPP reference database with a confidence limit of 95% (MD5: 7879792a6f42c5325531de9866f5c4de) [36, 37]. Mitochondria and Chloroplast features were filtered out via taxa filter-table. Low abundance and low prevalence ASV were filtered out for ANCOM analysis via feature-table filter-samples. Lastly, the feature table was filtered by *Salmonella* inocula, Typhimurium and Infantis, via feature-table filter-samples. The differential abundance between the phyla and genera was identified using ANCOM via q2-composition [38].

Alpha and Beta diversity analyses were rarified at 14,682 reads and 17,022 for *S*. Typhimurium and Infantis. The depths chosen were where read samples reached their maximum and sustained differences in diversity. Rarefication retained 572,598 (54.31%) features in 39 (90.70%) samples (Typhimurium: N = 39, n = 7–9, k = 5) and 612,792 (34.52%) features in 36 (87.80%) samples (Infantis: N = 36, n = 5–10, k = 5). Alpha diversity main effect and pairwise differences of treatment on Faith’s PD (phylogenetic richness) and Shannon’ Entropy (richness and evenness) were determined using Kruskal-Wallis tests [39, 40]. The main effect and pairwise differences on quantitative beta diversity indicators, such as Jaccard Dissimilarity [41] and Weighted Unifrac distance matrix [42, 43], were determined using ANOSIM. Microbiota main effects were considered significant if the main effect had P ≤ 0.05, and the pairwise effect had Q ≤ 0.05 with each statistical measurement within the QIIME2 pipeline. The Q-value represented the P-value adjusted for a strict false discovery rate and is incorporated into the QIIME-2.2023.2 pipeline.

## Results

### Effect of treatments on *Salmonella* Typhimurium and Infantis

**Fig 2:**
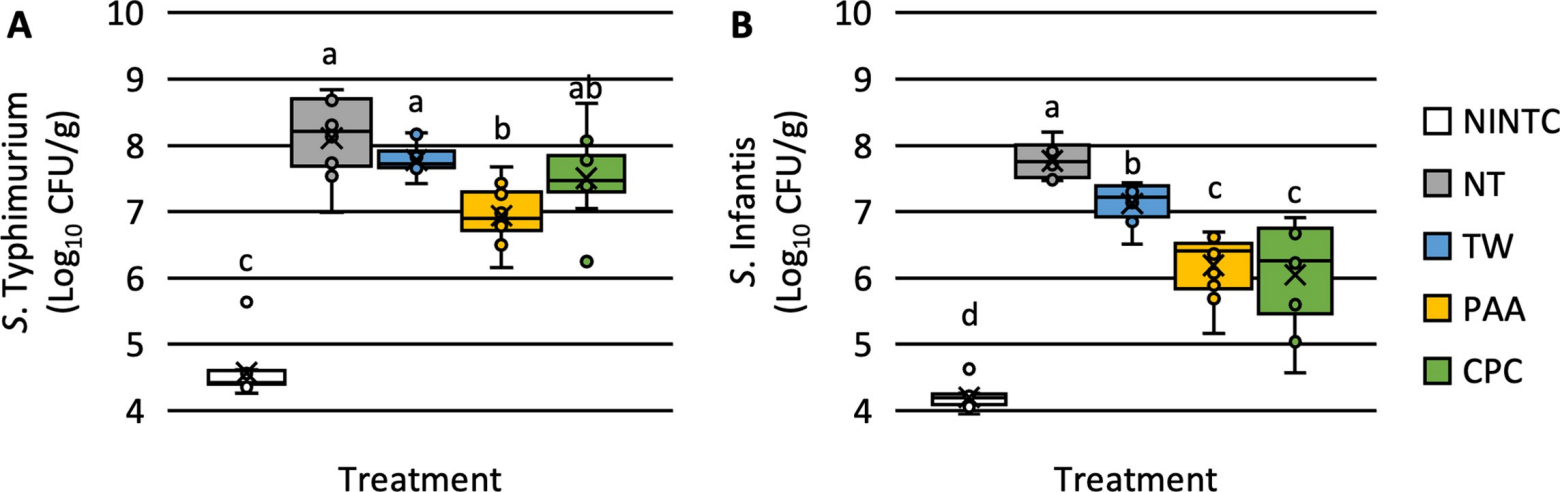
The effect of tap water (TW), peracetic (PAA), and cetylpyridinium chloride (CPC) on the firmly attached *Salmonella* Typhimurium (A) and Infantis (B). The skin of the *Pectoralis major* was cut into 4 × 4 cm squares that were inoculated and allowed to attach for 30 min at 4°C. Skins were removed and shaken for 30s to remove non-firmly attached *Salmonella*. Skins were treated for 20s in respective treatments and stomached for 1 min at 200 rpm and *Salmonella* was enumerated. Significance is denoted by different connecting letters^a-d^ (N = 50; n = 10; k = 5; P < 0.05).

There was a significant effect of treatment on the load of both *Salmonella* Typhimurium and Infantis (*S*. Typhimurium: P < 0.0001, N = 50, n = 10; *S*. Infantis: P < 0.0001, N = 50, n = 10; **Fig 2A**). The chicken skins contained a background level of 4.57 ± 0.12 (SEM) Log_10_ CFU/g of an NA-resistant *S*. Typhimurium as determined on the no inoculation no treatment control (NINTC). The only significant difference in the load of *S*. Typhimurium among treatments compared to the skins treated as NTC (8.11 ± 0.19 Log_10_ CFU/g) was those treated with 600 ppm PAA (6.94 ± 0.14 Log_10_ CFU/g). There was no difference between the skins treated as the NTC (8.11 Log_10_ CFU/g) and those treated with TW and CPC (7.78 ± 0.07 and 7.51 ± 0.20 Log_10_ CFU/g).

When inoculated with *S*. Infantis, the chicken skins possessed a background presence of 4.20 ± 0.18 Log_10_ CFU/g of an NA-resistant *S*. Infantis as demonstrated via the no inoculation no treatment control (NINTC; **Fig 2B**). The use of both antimicrobial treatments, PAA (6.19 ± 0.15 Log_10_ CFU/g) and CPC (6.05 ± 0.25 Log_10_ CFU/g), on poultry skins resulted in lower concentration of *S*. Infantis compared to NTC and TW (7.77 ± 0.08 and 7.12 ± 0.10 Log_10_ CFU/g). Those treated with CPC were not different from those treated with PAA.

### Impact of treatments on *hilA* expression

**Fig 3:**
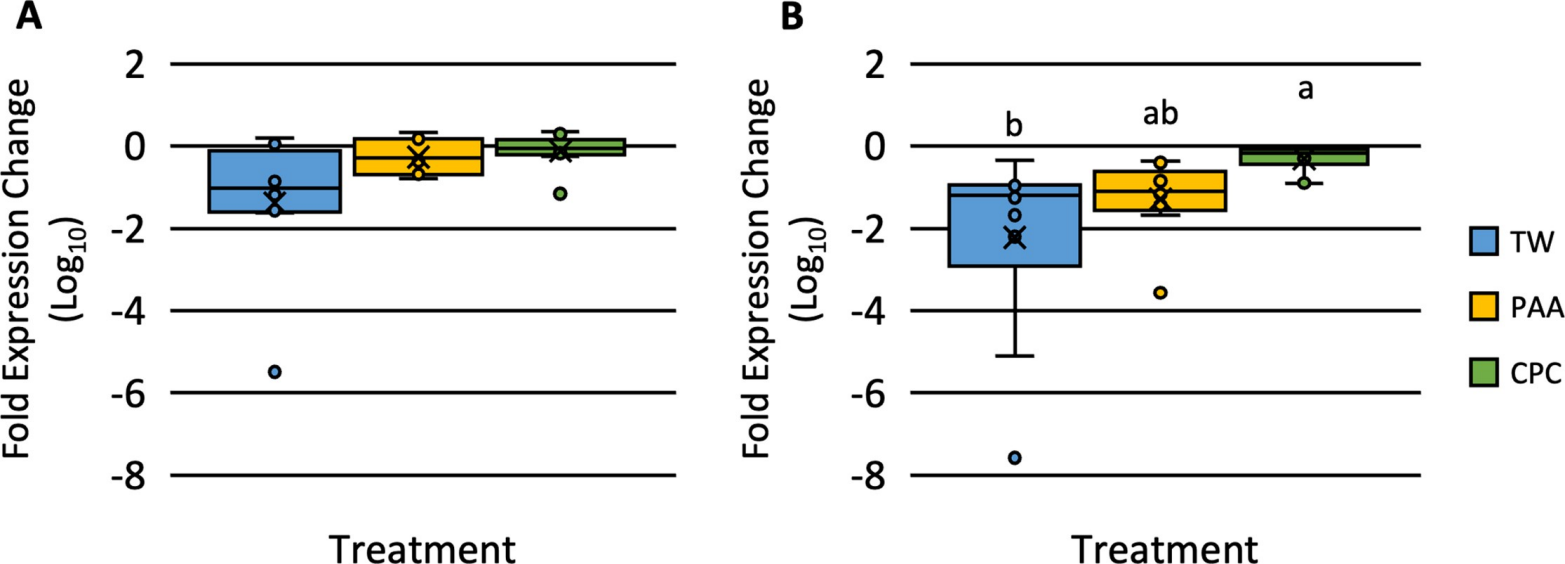
The expression of *hilA* of *Salmonella* Typhimurium (A) and *S*. Infantis (B) of the skin of the *Pectoralis major* of chicken treated with tap water (TW), peracetic acid (PAA), and cetylpyridinium chloride (CPC) relative to the no treatment control (NTC). The skin of the *Pectoralis major* was cut into 4 × 4 cm squares that were inoculated and allowed to attach for 30 min at 4°C. Skins were removed and shaken for 30s to remove non-firmly attached *Salmonella*. Skins were treated for 20s in respective treatments and stomached for 1 min at 200 rpm and the expression of *hilA* was determined via qPCR (A: N = 50, n = 10, k = 5; P < 0.05; B: N = 50, n = 10, k = 5; P > 0.05). Significance is denoted by different connecting letters^a-b^.

There was no effect of treatment on the expression of *hilA* Fold Expression Change over 16S when *S*. Typhimurium was inoculated (P = 0.0598; **Fig 3A**). There was an effect of treatment on the fold expression change of *hilA* over total 16S when *S*. Infantis inoculated on chicken skin (P = 0.0252; **Fig 3B**). Those inoculated with *S*. Infantis and treated with CPC (-0.308 Log_10_ Fold Expression Change) had a greater fold expression change of *hilA* compared to those treated with TW (-2.218 Log_10_ Fold Expression Change). Those treated with PAA (-1.288 Log_10_ Fold Expression Change) were not different than those treated with TW (-2.218 Log_10_ Fold Expression Change).

#### Skin microbiota response to treatments when inoculated with *Salmonella* Typhimurium

**Fig 4:**
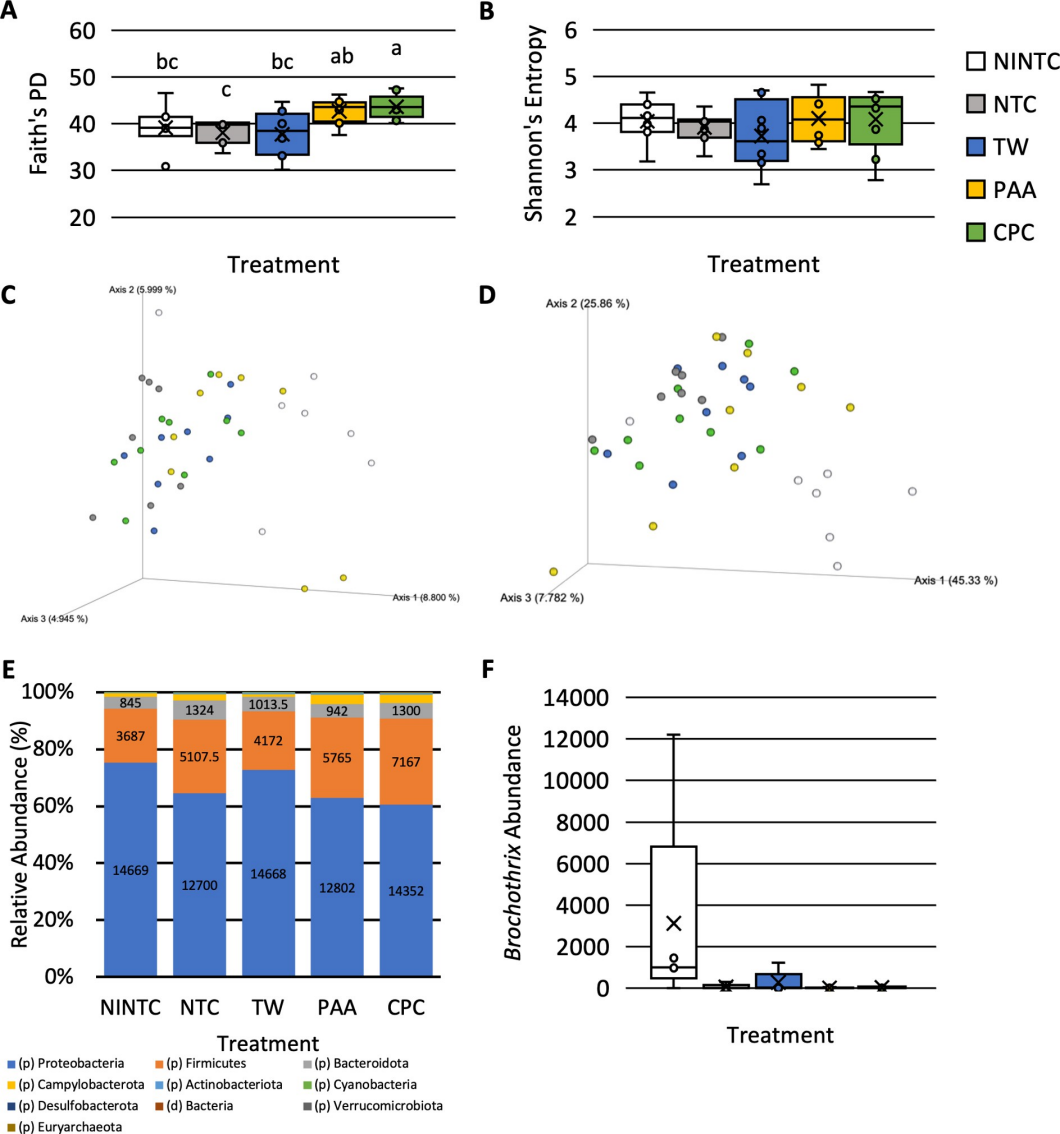
Impact of treatment, no inoculated no treatment control (NINTC), no treatment control (NTC), tap water (TW), TW + 0.5% cetylpyridinium chloride (CPC), and 600 ppm peracetic acid (PAA) on the skin microbiota of the pectoralis major of commercial broilers when inoculated with *S*. Typhimurium. The alpha, Faith’s PD (**A**) and Shannon’s Entropy (**B**), and beta, Jaccard (**C**) and Weighted Unifrac (**D**), diversity and the significant phyla (**E**) and genera (**F**) of the chicken skin inoculated with *Salmonella* Typhimurium (N = 39, n = 7–9; k = 5). The only significant genera were Brochothrix (W = 118).

As previously stated, the inoculation of poultry pectoralis major skin with *Salmonella* Typhimurium served as the control state. When the chicken skin was inoculated with *S*. Typhimurium, there was an effect of antimicrobial treatments on the diversity and composition of the firmly attached microbiota present on the skin (P < 0.05; **Fig 4**). There was an effect of antimicrobial treatment on the phylogenetic diversity of treated skins (Faith’s PD; P < 0.05; Q < 0.05). Those treated with CPC had higher phylogenetic diversity than those treated as the NINTC, NTC, or treated with TW (Q < 0.0.5; **Fig 4A**). There were no differences in the phylogenetic diversity (Faith’s PD) within those treated with PAA and CPC (Q > 0.05). Unlike Faith’s PD, there was no effect on the overall richness and evenness (Shannon’s Entropy; P > 0.05; **Fig 4B**).

There was an effect of treatment on both the abundance (Jaccard Dissimilarity) and weighted phylogenetic differences (Weighted Unifrac; P < 0.05; **Fig 4C and 4D** and **S1 Table**). There was a difference between the Jaccard Dissimilarity of those treated as NINTC and those treated as NTC and those treated with PAA and CPC (Q < 0.05; **Fig 4C**). There were trending differences between those treated with TW and those treated with NTC, PAA, and CPC (0.10 < Q > 0.05). Similar to Jaccard, there were differences between the weighted phylogenetic differences (Weighted Unifrac) between those treated as NINTC and those treated as NTC or treated with TW, PAA, and CPC (Q < 0.05; **Fig 4D**). In addition, there was a difference in the Weighted Unifrac between those treated as NTC and those treated with TW (Q < 0.05).

The primary composition of the microbiota of skins inoculated with *S*. Typhimurium at the phyla level consisted of Proteobacteria, Firmicutes, Bacteroidota, Campylobacterota, Cyanobacteria, and Actinobacteriota, with Proteobacteria comprising more than 60% of the composition and Firmicutes being between 20 and 30% of composition (**S1A Fig**). All phyla were differentially abundant as determined by ANCOM (P < 0.05; **Fig 4E**). Proteobacteria were differentially abundant than two other taxa at the phyla level and Actinobacteriota were differentially abundant than 1 other taxa at the phyla level (P < 0.05). All other taxa at the phyla level, although significantly, were not differentially abundant to any other taxa (W = 0; P < 0.05).

At the genera level, the compositions between those treated as the NINTC and those treated with NTC, TW, PAA, and CPC were more distinct with those treated as NINTC having a higher relative abundance of *Pseudomonas* and the inoculated treatments having a higher relative abundance of *Enterobacterales* (**S1B Fig**). The only significant genera at the genus level were *Brochothrix* which was abundantly different than 118 other taxa (P < 0.05; W = 118; **Fig 4F**). Those treated as the NINTC had the highest relative abundance of *Brochothrix*.

#### Skin microbiota response to treatments when inoculated with *Salmonella* Infantis

**Fig 5:**
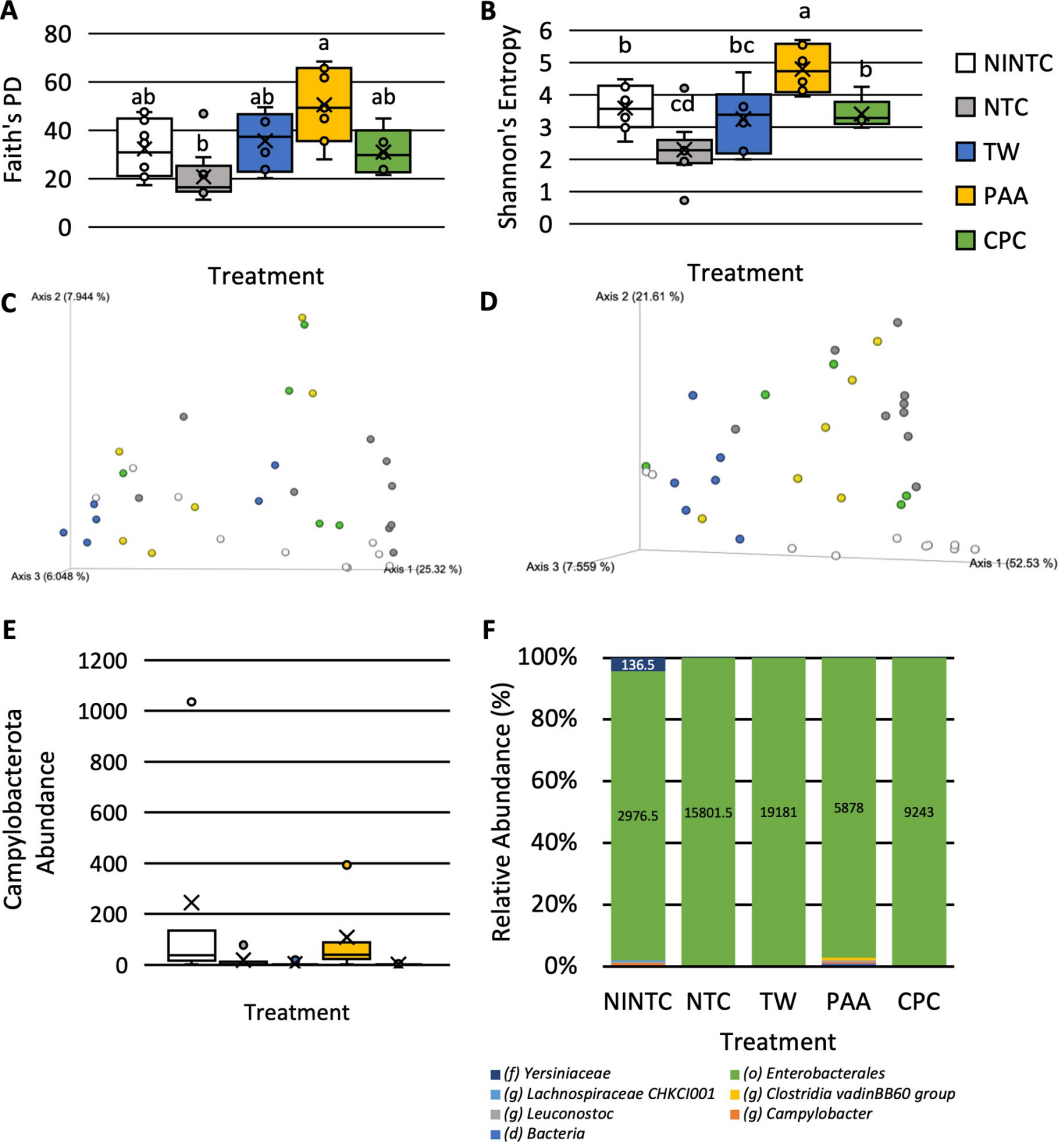
Impact of treatment, no inoculated no treatment control (NINTC), no treatment control (NTC), tap water (TW), TW + 0.5% cetylpyridinium chloride (CPC), and 600 ppm peracetic acid (PAA) on the skin microbiota of the pectoralis major of commercial broilers when inoculated with *S*. Infantis. The alpha, Faith’s PD (**A**) and Shannon’s entropy (**B**), and beta, Jaccard (**C**) and Weighted Unifrac (**D**), diversity and the significant phyla (**E**) and genera (**F**) of the chicken skin inoculated with *Salmonella* Infantis (N = 36, n = 5–10; k = 5). Campylobacterota was the only significantly different taxa at the phyla level (W = 7). At the genus level, *Yersiniaceae*, *Enterobacterales*, *Lachnospiraceae CHKCI001*, *Clostridia vadinBB60 group*, *Leuconostoc*, *Campylobacter*, and bacteria were different (W = 13, 18, 8, 16, 17, 40, 8).

The treatments significantly impacted the diversity and composition of the microbiota when inoculated with *S*. Infantis. Those treated with PAA had a greater phylogenetic diversity (Faith’s PD) and a greater richness and evenness (Shannon’s Entropy) than those treated as NTC (**Fig 5A and 5B**; P < 0.05; Q < 0.05). The phylogenetic diversity within those treated as NINTC or with TW and CPC was not different from those treated with PAA (Q < 0.05; **Fig 5A**). The richness and evenness (Shannon’s Entropy) of those treated with NINTC and CPC were not different from TW but were different from PAA and NTC (Q < 0.05; **Fig 5B**). Those treated with NTC did not differ in richness and evenness than those treated with TW (Q > 0.05).

There was a significant effect of treatment on the beta diversity of *S*. Infantis inoculated chicken skins (**Fig 5C and 5D** and **S1 Table**; P < 0.05). The Jaccard Dissimilarity between those treated as NINTC and those treated with NTC, TW, and PAA were different (**Fig 5C**; Q < 0.05). The Jaccard Dissimilarity between those treated with PAA was different from that of those treated with NTC, TW, and CPC (Q < 0.05). The dissimilarity between those treated with CPC was different from those treated with NTC and PAA but not NINTC or TW. The dissimilarity between those treated with TW tended to be different from those treated with NTC (0.10 < Q > 0.05). The phylogenetic diversity between those treated with NTC and PAA was different (Q < 0.05) with PAA tending to be different from those treated with NINTC, NTC, and CPC (0.10 < Q > 0.05; **Fig 5D**). The phylogenetic diversity between those treated with TW tended to be different from those treated with NINTC and NTC (0.10 < Q > 0.05).

Compositionally at the phyla level, skins inoculated with *S*. Infantis consisted of Proteobacteria, Firmicutes, and Bacteroidota (**S2A Fig**). The relative abundance of those treated with CPC more closely resembled the NINTC group than the remaining antimicrobial treatments. As well, at the phyla level, Campylobacteriota was the only significantly different abundant taxa being abundantly different than 7 other taxa (W = 7; **Fig 5E**). Although Campylobacterota was a small portion of the composition, its abundance was greater in those treated as the NINTC and those treated with PAA.

At the genus level, the compositions of treated skins were comprised of 141 different genera with *Enterobacterales*, *Pseudomonas*, and *Enterobacteriaceae* being greater proportions of the compositions (**S2B Fig**). *Yersiniaceae*, *Enterobacterales*, *Lachnospiraceae CHKCI001*, *Clostridia vadinBB60 group*, *Leuconostoc*, *Campylobacter*, and bacteria were differentially abundant at the genus level. (W = 13, 18, 8, 16, 17, 40, 8; **Fig 5F**). *Yersiniaceae* was more prevalent in those treated as NINTC and *Enterobacterales* was least prevalent among those treated with CPC. *Campylobacter* was more prevalent among those treated as NINTC or with PAA than in other treatments.

## Discussion

In the poultry industry, the use of PAA in harvest and processing practices easily outnumbers the use of any other antimicrobial intervention. As early as 2011, it has been documented that PAA has been the predominant post-chill antimicrobial intervention in the US poultry industry [10]. Ebel et al. [44] reported that out of the 167 poultry establishments surveyed between July 30, 2016 and December 18, 2018 that 74% (124 of 167) and 87% (146 of 167) of the establishments used PAA exclusively as the carcass and parts antimicrobial intervention, respectively. Antimicrobial alternative CPC was used in 14% (23 of 167) and 4% (7 of 167) of the establishments as the carcass and parts antimicrobial intervention [44].

Although the use of PAA within the poultry industry has demonstrated widespread efficacy compared to other antimicrobial interventions [45], the widespread reliance of the industry on one specific antimicrobial, PAA, is bound to cause resistance and the persistence of specific pathogens. More specifically, there is a concern that the use of organic acids, such as PAA, for the decontamination of animal processing-associated matrices can result in the selection or development of acid-tolerant strains that are more likely to survive the gastric environment and develop tolerance to stressors such as heat, salts, and osmosis [46, 47]. Furthermore, Berk et al. [48] found a correlation between acid resistance and pathogenicity which can be explained by the exposure of *Salmonella* spp. to short-chain fatty acids (SCFA) such as acetate which induces the expression of the *Salmonella* pathogenicity island 1 (SPI-1) [47, 49, 50]. In addition, *Salmonella* tolerance to acids differs between strains and serovars [15, 48, 51]. For instance, Koyuncu et al. [51] showed that *S*. Infantis isolated from animal feed was more acid-tolerant than *S*. Putten, *S*. Senftenberg, and *S*. Typhimurium to a group of various acids. Thus, acid treatment of *Salmonella* decreases viable pathogen numbers but does not completely eliminate the pathogen [51]. For this reason, acid application for decontamination of *Salmonella* must be successful even against less susceptible strains.

### CPC is more effective against *Salmonella* Infantis than *S*. Typhimurium

There have been several publications demonstrating the use of CPC against *S*. Typhimurium on poultry products with varying results [10, 52–55]. Chen et al. [10] demonstrated improved efficacy of (0.07 and 0.1%) PAA versus (0.35 and 0.6%) CPC in reducing *S*. Typhimurium (isolated from University of Auburn Poultry Research Unit; 35 μg/mL NA-resistant) in ground chicken meat. When treating *Salmonella* inoculated (*S*. Montevideo, Typhimurium, Heidelberg, Enteritidis, and Newport resistant to 20 μg/mL of NA and 25 μg/mL of novobiocin isolated from poultry origin) chicken wings with 700 ppm PAA for 20 s and 0.4% CPC for 10 s, PAA was more effective in mitigating *Salmonella populations* at 0 and 24 h post-treatment (PAA: 4.0 and 3.8 Log_10_ CFU/mL; CPC: 4.7 and 4.8 Log_10_ CFU/mL). Moore et al. [53] investigated the effect of various antimicrobial treatments against *S*. Heidelburg (NA 19 isolated from retail ground turkey resistant to NA 60 μg/mL) inoculated chicken frames and demonstrated the submersion of the frames for 10-s in 0.6% CPC was not statistically different than the submersion for 10-s in 1% PAA at 0 and 24 h post-treatment (CPC: 0.5 and 0.9 Log_10_ CFU/g reduction compared to sterile DI water; PAA: 0.9 and 1.4 Log_10_ CFU/sample reduction compared to sterile DI water). The use of 0.35 and 0.60% CPC (∼2.5 and 3.5 Log_10_ CFU/g reduction compared to water) in a post-chill decontamination tank for 23 s resulted in the greatest efficacy compared to the use of 0.07 and 0.1% PAA (∼1.5 Log_10_ CFU/g reduction compared to water) on controlling an isolate of *S*. Typhimurium (isolated from a chicken house at the University of Auburn Poultry Research Unit and resistant to 35 μg/mL of NA) of poultry origin [54]. Applied as 10, 20, and 30 s immersion dips on chicken drumsticks, the reduction of inoculated *S*. Typhimurium of poultry origin was greater with the application of 0.35 and 0.6% CPC compared to 0.07 and 0.1% PAA with 0.6% CPC with a 30 s contact being the most effective treatment [55]. In summation, the efficiency of CPC against attached *Salmonella* depends on its concentration and time of exposure [56].

However, there are limited reports on the response of *S*. Infantis to the use of CPC on poultry carcasses, parts, and skin [21]. With evidence of varying acid-tolerance between *S*. Infantis and other serovars, it is important to also understand the effect of antimicrobial interventions on more persistent strains surviving current poultry practices such as the widespread use of PAA as an antimicrobial intervention. In recent research, Wythe et al. [21] compared the efficacy of PAA to CPC on bone-in, skin-on chicken thighs and demonstrated the application of 0.5% CPC (4.29 Log_10_ CFU/g) to have greater efficacy against *S*. Infantis compared to PAA (4.96 Log_10_ CFU/g) 24 h post-treatment as CPC was the only treatment that resulted in significantly less *S*. Infantis compared to TW (5.71 Log_10_ CFU/g). In the current study, different efficacies of CPC and PAA on *Salmonella* serovars Typhimurium and Infantis were observed with CPC being more effective against *S*. Infantis. The application of the surfactant, 0.5% CPC, on the individual chicken skin coupons for 20 s at room temperature did not produce statistically different results from 600 ppm PAA when the skins were inoculated with *S*. Typhimurium or *S*. Infantis. However, when the skins were inoculated with *S*. Infantis, those treated with CPC had the lowest *S*. Infantis load overall, besides the no-inoculated control, and were different from those treated with TW.

### CPC reduces the expression of *hilA* of *Salmonella* Infantis

As previously mentioned, it is known that different *Salmonella enterica* serovars possess different acid tolerance [15, 48, 51], with *S*. Infantis demonstrating a greater acid resistance than *S*. Putten, *S*. Senftenberg, and *S*. Typhimurium to various SCFA combinations (Koyuncu et al., 2013). It has also been established that acetate is known to induce the expression of SPI-1 gene expression [47, 49, 50]. Hamed et al. [57] demonstrated that acetate and nutrients (yeast extract) introduced individually and in combination with *Salmonella* Typhimurium ATCC 14028 cultures induced the expression of the SPI-1 gene of *S*. Typhimurium. This induced expression of SPI-1 by acetate is regulated by the transcription factors BarA/SirA, HilD, and subsequently HilA, which activate *hilA* and SPI-1 expression [58]. With the degradation of aqueous solutions of PAA to acetic acid and hydrogen peroxide [59], there is a concern for the widespread use of PAA in the poultry industry to cause increased resistance and pathogenicity in persistent and emerging *Salmonella* serovars such as *S*. Typhimurium and *S*. Infantis.

There was no difference in the expression of *hilA* between the treatments when *S*. Typhimurium was inoculated, but when *S*. Infantis was inoculated, there was a difference in the expression of *hilA* of those treated with CPC compared to those treated with TW. It has been demonstrated in mouse models, that *S*. Typhimurium can invade deeper sub-epithelial cecal tissue in comparison to *S*. Infantis which exclusively only enters epithelial tissue [60]. Although the results demonstrated by Braukmann et al. [60] may be more applicable to a live host, in the current study, this might provide a potential explanation of why a lipid-soluble surfactant, CPC, could decrease *S*. Infantis counts on chicken skin and alter the expression of *hilA* gene.

It is important to note that the reductions in bacteria did relate to the cycle threshold (quantity) of the total 16S present within a sample. As the efficacy of the chemical interventions on the chicken skin increased, *Salmonella* and total bacterial populations decreased, as seen with CPC on *Salmonella* Infantis. In response, the quantity of total 16S was reduced. In future studies, it is advised to use an internal control gene for *Salmonella* qPCR assays where the reduction of the total populations of bacteria does not impact the quantity of the control gene, such as was seen in the current study. As such, a control gene such as the *rsmC* gene of *Salmonella* Typhimurium should be utilized to map the response of *Salmonella* virulence when comparing poultry processing chemical interventions [61]. Additionally, as the *invA* gene has been demonstrated to represent the detection of only live *Salmonella* among food products [62], it can be used as an additional control in future studies. Regardless, this study allows us to compare the impact of chemical interventions on the virulence of firmly attached *Salmonella* on poultry skin. In the current study, among those inoculated with *S*. Infantis, those treated with CPC had a more similar expression to those treated with the NTC (experimental control condition) with a fold expression change of -0.308 Log_10_.

### *S*. Typhimurium and Infantis inoculated chicken skin microbiota

It has been generally understood that the structure of poultry skin is complex making the surface more difficult to clean and mitigate bacteria attachment and invasion during harvest and processing practices [2–6]. Therefore, poultry skin is more susceptible to bacterial contamination. Dominant phyla of poultry skin after chilling include Proteobacteria, Firmicutes, Bacteroidetes, and Actinobacteria [63–66]. At the genera level, *Acinetobacter*, *Pseudomonas*, *Lactobacillus*, *Streptococcus*, *Staphylococcus*, and *Chryseobacterium* dominate the chicken skin [63–66]. Within the feather follicle, the dominant genera after chilling include *Acinetobacter*, *Psychrobacter*, *Macrococcus*, *Aeromonas*, *Comamonas*, *Acidovorax*, *unidentified Enterobacteriaceae*, *Pseudomonas*, *Arcobacter*, *Uruburuella*, *Kurthia*, *Enterococcus*, *Vitreoscilla*, and *Enhydrobacter* [67].

The current study’s results align with recent research delineating the dominant phyla and genera seen among poultry carcasses and skin obtained after immersion chilling. In the current study, regardless of the inocula used, *S*. Typhimurium or Infantis, the dominant phyla were primarily Proteobacteria, Firmicutes, and Bacteroidota among the NINTC controls. However, among those inoculated with *S*. Typhimurium, Actinobacteriota, Cyanobacteria, and Campylobacterota were also among the dominant phyla (>1%). Across both serovars, the dominant genera were primarily *Pseudomonas* and *Enterobacterales*, with several other genera being less dominant.

### Influence of CPC and PAA chicken skin microbiota when inoculated with *S*. Typhimurium and Infantis

There is limited research regarding the effect of poultry processing antimicrobial aids on the broiler carcass skin microbiota when different serovars of *Salmonella* infect the carcass. In the current study, the authors utilized both *S*. Typhimurium (control serovar) and Infantis (acid-resistant serovar) to demonstrate the efficacy of an organic acid, PAA, and a surfactant, CPC, on reducing *Salmonella* and their subsequent effects on the skin microbiota. The current research demonstrates the differential response of the microbiota to varying poultry processing aids, PAA and CPC, when inoculated with *S*. Typhimurium or *S*. Infantis.

One of the major differences between the microbiota effects of PAA and CPC, when inoculated with *S*. Typhimurium versus *S*. Infantis, is that there were no differences in the microbiota diversity between the two chemical processing aids when skins were inoculated with *S*. Typhimurium. However, when skins were inoculated with *S*. Infantis, there were differences or trending differences between those treated with PAA and CPC with PAA having a greater phylogenetic diversity and richness and evenness than CPC. In addition, CPC was not different from those treated as NINTC potentially indicating that the microbiota of those treated with CPC were able to return to a non-infected state. Previously, Wythe et al. [21] reported similar findings when investigating the effects of 0.5% CPC and 600 ppm PAA on the levels of *S*. Infantis and corresponding microbiota of skin-on, bone-on chicken thighs. Although, Wythe et al. [21] demonstrated there were no differences between the diversity (Faith’s PD, Shannon’s Entropy, and Jaccard) between PAA and CPC in trial 2, they also did not have differences between CPC and the NINTC as was demonstrated in the current study.

### Selectivity of acid tolerant microbiota among PAA-treated skin when inoculated with *S*. Infantis

The higher diversity of those treated with PAA, when inoculated with S. Infantis, can be explained through the taxonomic bar charts and the results of ANCOM. When inoculated with *S*. Infantis, those treated with PAA has a greater relative abundance of taxa belonging to *Ruminococcaceae*, *Oscllibacter*, *Lactobacillus*, *Lachnspiraceae*, and *Bacteroides* than other treatments providing a more diverse community. This observation was not seen when skin was inoculated with *S*. Typhimurium. In addition, at the genus level when inoculated with *S*. Infantis, the abundance of *Yersiniaceae*, *Enterobacterales*, *Lachnospiraceae CHKCI001*, *Clostridia vadinBB60 group*, *Leuconostoc*, *Campylobacter*, and bacteria was different (W = 13, 18, 8, 16, 17, 40, 8). Although no pH measurements were taken on the treated skin, a proportion of significant genera that were short-chain fatty acid-producing bacteria such as *Lachnospiraceae CHKCI001*, *Clostridia vadinBB60 group*, and *Leuconostoc*. The significance of these taxa could be due to 1) the acid-resistant nature of *S*. Infantis and 2) the potential of PAA, an organic acid, to select for acid-tolerant microorganisms.

When inoculated with Typhimurium, all were significant at the phyla level, but the only significant genera were *Brochothrix* (W = 118). The *Brochothrix* genus was significantly associated with all the treatments and was most abundant in the NINTC group. *Brochothrix* is a common spoilage organism of meat products stored at chilled temperatures [68].

### Potential relationship between *S*. Infantis and *Campylobacter*

In the current study, when skins were inoculated with *S*. Infantis, Campylobacterota was different at the phyla level, and *Campylobacter* was among the significant taxa at the genus level. Previous research demonstrates a positive relationship between *Campylobacter* and *Salmonella* colonization within live broiler flocks [69]. Although the current study explored the microbiota response of broiler carcass skin, a relationship between *S*. Infantis and *Campylobacter* was demonstrated. Thus, future experiments should incorporate the monitoring effects of antimicrobials on both *Campylobacter* and *Salmonella* serovars.

## Conclusion

Isolated skins treated with 600 ppm PAA and 0.5% CPC were not statistically different in the current study. However, when the isolated skins were inoculated with *S*. Infantis, those treated with CPC possessed the lowest load overall, besides the no-inoculated control and were significantly different than those treated with TW. There was no effect of treatment on the expression of *hilA* when skins were inoculated with *S*. Typhimurum; however, there was a difference in the expression of *hilA* of those treated with CPC compared to those treated with TW when inoculated with *S*. Infantis. Also, there were distinct differences in the microbiota diversity and composition due to inocula and treatment. Understanding the influence that specific *Salmonella* serovars have on the skin microbiota will be important in determining the most effective antimicrobial in terms of mitigating pathogens and spoilage microorganisms.

Surfactants, such as CPC, increase the solubility of chemicals and their penetration [70]. Therefore, further research should concentrate on the combinatorial effect of organic acids with CPC on chicken skin, as previous studies performed by Kim and Day [71] and Dittoe et al. [29] showed synergistic antimicrobial qualities between various antimicrobials in combination with surfactants that possibly are able to reduce the integumentary buffering effect and avert the emergence of acid-tolerant bacteria. In conclusion, the treatment of skins with 0.5% CPC decreased the concentration of *Salmonella* Infantis and the expression of *hilA*, a gene involved in the activation of invasion genes. Although no key differences between the skin microbiota of those treated with PAA and CPC occurred when skins were inoculated with *S*. *Typhimurium*, when inoculated with *S*. Infantis, CPC-treated skins maintained a diversity similar to that of the no-inoculated, no-treated controls.

## Supporting information

S1 Fig Effect of the treatment on the skin microbiota of the pectoralis major of commercial broilers when inoculated with *S*. Typhimurium. The median taxa abundance at the phylum (**A**) and genus (**B**) levels. Taxa with a median abundance equal to or less than 1% of the total population was considered as “Other”.
(TIF)

S2 Fig Effect of the treatment on the skin microbiota of the pectoralis major of commercial broilers when inoculated with *S*. Infantis. The median taxa abundance at the phylum (**A**) and genus (**B**) levels. Taxa with a median abundance equal to or less than 1% of the total population was considered as “Other”.
(TIF)

S1 Table Pairwise treatment differences from alpha and beta diversity metrics of chicken pectoralis major skins inoculated with *Salmonella* Typhimurium and Infantis. (DOCX)
