# Supplementary figures and images for: Mitigating the attachment of Salmonella Infantis on isolated poultry skin with cetylpyridinium chloride
Source: PLoS One. 2023 Dec 21;18(12):e0293549. doi: 10.1371/journal.pone.0293549 (PMC10735015; doi:10.1371/journal.pone.0293549)

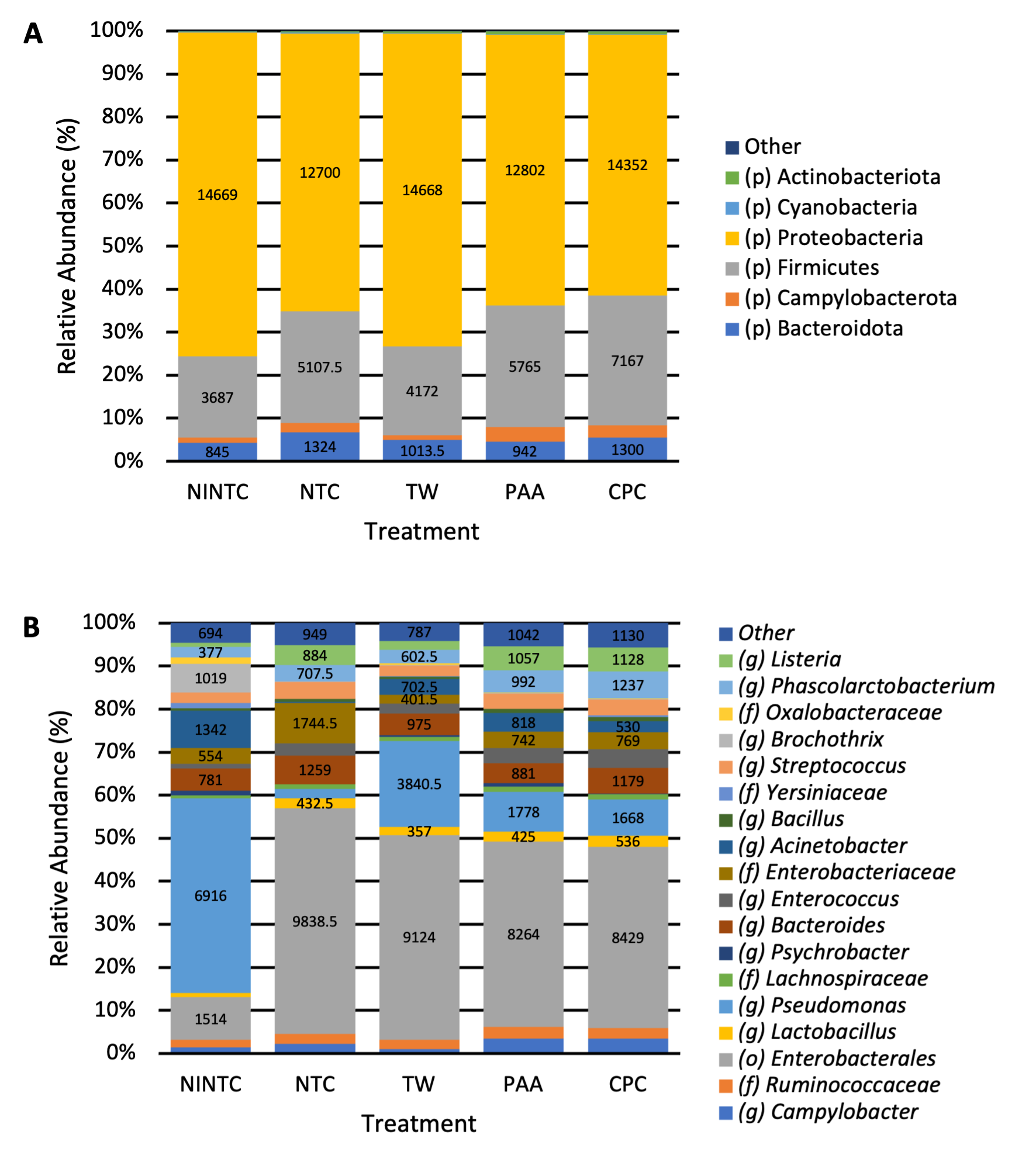

Supplement: S1 Fig — The median taxa abundance at the phylum (A) and genus (B) levels. Taxa with a median abundance equal to or less than 1% of the total population was considered as “Other”. (TIF) [file pone.0293549.s001.tif]

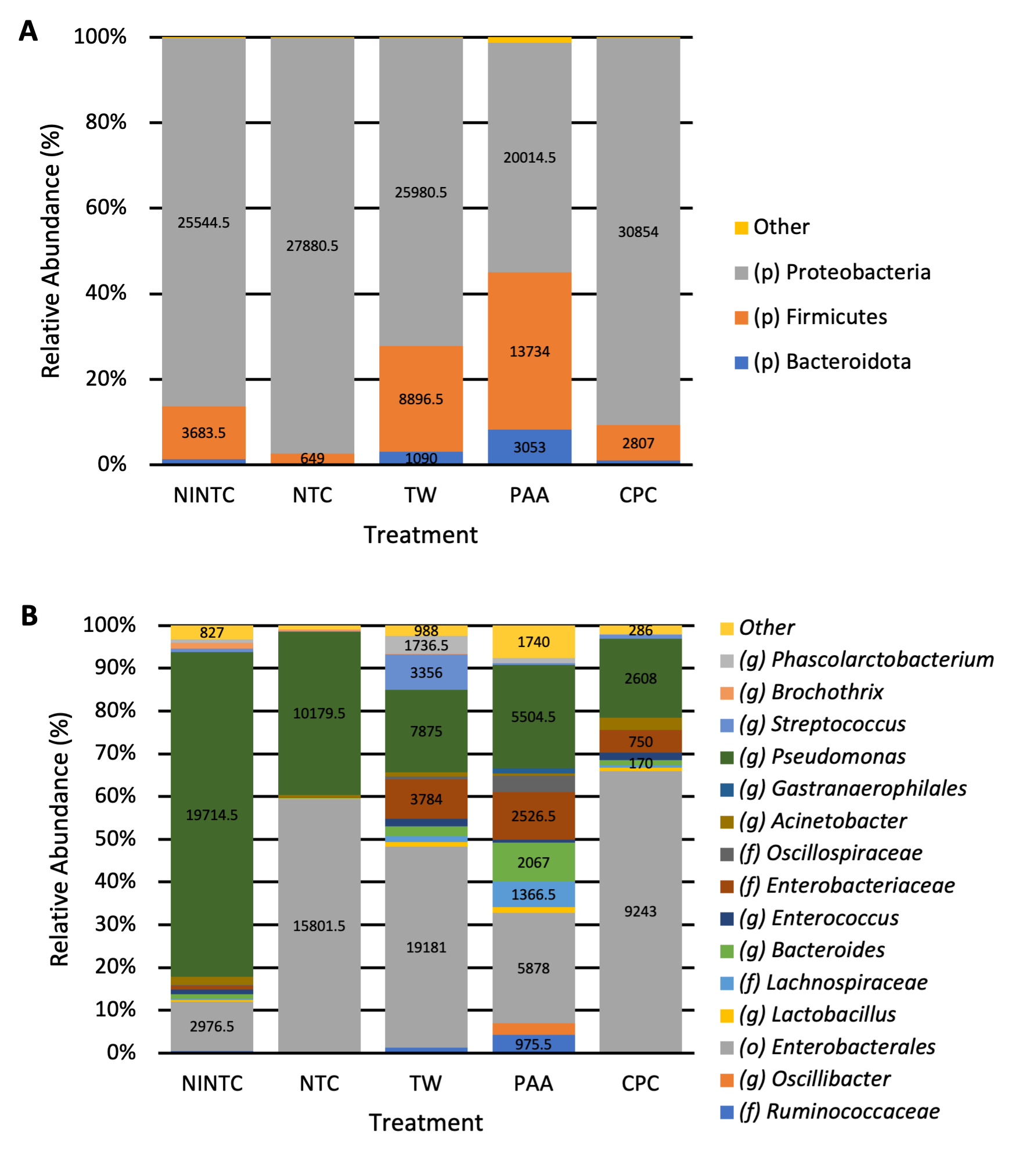

Supplement: S2 Fig — The median taxa abundance at the phylum (A) and genus (B) levels. Taxa with a median abundance equal to or less than 1% of the total population was considered as “Other”. (TIF) [file pone.0293549.s002.tif]
