# Supplementary material for: Mitigating the attachment of Salmonella Infantis on isolated poultry skin with cetylpyridinium chloride
Source: PLoS One. 2023 Dec 21;18(12):e0293549. doi: 10.1371/journal.pone.0293549 (PMC10735015; doi:10.1371/journal.pone.0293549)
Supplement: S1 Table — (DOCX) [file pone.0293549.s003.docx]

**Supplemental Table 1.** Pairwise treatment differences from alpha and beta diversity metrics of chicken pectoralis major skins inoculated with *Salmonella* Typhimurium and Infantis.

|  |  |  | Alpha Diversity^2^ | | | | | | Beta Diversity^3^ | | | | | |
| --- | --- | --- | --- | --- | --- | --- | --- | --- | --- | --- | --- | --- | --- | --- |
|  |  |  | Faith's PD | | | Shannon's Entropy | | | Jaccard | | | Weighted Unifrac | | |
| Inocula | Treatment | Treatment | H^4^ | P-value | Q-value | H | P-value | Q-value | R^5^ | P-value | Q-value | R | P-value | Q-value |
| *S.* Typhimurium | CPC (n = 9) | NINTC (n = 7) | 5.672 | 0.017 | **0.043** | 0.070 | 0.791 | 0.879 | 0.460 | 0.003 | **0.020** | 0.504 | 0.003 | **0.015** |
|  |  | NTC (n = 7) | 9.751 | 0.002 | **0.018** | 1.011 | 0.315 | 0.834 | 0.036 | 0.387 | 0.387 | 0.072 | 0.195 | 0.217 |
|  |  | PAA (n = 8) | 0.148 | 0.700 | 0.908 | 0.148 | 0.700 | 0.875 | 0.046 | 0.297 | 0.330 | -0.076 | 0.818 | 0.818 |
|  |  | TW (n = 8) | 5.787 | 0.016 | **0.043** | 0.454 | 0.501 | 0.834 | 0.138 | 0.049 | ***0.083*** | 0.111 | 0.117 | 0.146 |
|  | NINTC (n = 7) | NTC (n = 7) | 0.037 | 0.848 | 0.908 | 0.690 | 0.406 | 0.834 | 0.522 | 0.006 | **0.020** | 0.595 | 0.005 | **0.017** |
|  |  | PAA (n = 8) | 2.625 | 0.105 | 0.175 | 0.000 | 1.000 | 1.000 | 0.507 | 0.005 | **0.020** | 0.532 | 0.002 | **0.015** |
|  |  | TW (n = 8) | 0.121 | 0.728 | 0.908 | 1.339 | 0.247 | 0.834 | 0.115 | 0.127 | 0.159 | 0.267 | 0.011 | **0.022** |
|  | NTC (n = 7) | PAA (n = 8) | 6.482 | 0.011 | **0.043** | 0.214 | 0.643 | 0.875 | 0.123 | 0.112 | 0.159 | 0.133 | 0.084 | 0.127 |
|  |  | TW (n = 8) | 0.013 | 0.908 | 0.908 | 0.656 | 0.418 | 0.834 | 0.200 | 0.033 | ***0.083*** | 0.249 | 0.007 | **0.018** |
|  | PAA (n = 8) | TW (n = 8) | 4.412 | 0.036 | *0.071* | 1.335 | 0.248 | 0.834 | 0.156 | 0.050 | ***0.083*** | 0.100 | 0.089 | 0.127 |
|  |  |  |  |  |  |  |  |  |  |  |  |  |  |  |
| *S.* Infantis | CPC (n = 5) | NINTC (n = 10) | 0.000 | 1.000 | 1.000 | 0.135 | 0.713 | 0.793 | 0.159 | 0.106 | 0.118 | 0.162 | 0.095 | 0.136 |
|  |  | NTC (n = 9) | 3.738 | 0.053 | *0.089* | 6.084 | 0.014 | **0.029** | 0.283 | 0.036 | **0.051** | 0.114 | 0.162 | 0.180 |
|  |  | PAA (n = 6) | 4.033 | 0.045 | *0.089* | 5.633 | 0.018 | **0.029** | 0.445 | 0.007 | **0.014** | 0.341 | 0.025 | *0.064* |
|  |  | TW (n = 6) | 0.533 | 0.465 | 0.582 | 0.033 | 0.855 | 0.855 | -0.032 | 0.503 | 0.503 | 0.079 | 0.232 | 0.232 |
|  | NINTC (n = 10) | NTC (n = 9) | 5.607 | 0.018 | *0.063* | 9.127 | 0.003 | **0.016** | 0.412 | 0.001 | **0.005** | 0.120 | 0.062 | 0.103 |
|  |  | PAA (n = 6) | 5.188 | 0.023 | *0.063* | 6.224 | 0.013 | **0.029** | 0.468 | 0.004 | **0.010** | 0.297 | 0.022 | *0.064* |
|  |  | TW (n = 6) | 0.188 | 0.664 | 0.738 | 0.576 | 0.448 | 0.560 | 0.259 | 0.022 | **0.037** | 0.226 | 0.032 | *0.064* |
|  | NTC (n = 9) | PAA (n = 6) | 8.000 | 0.005 | **0.047** | 8.681 | 0.003 | **0.016** | 0.869 | 0.001 | **0.005** | 0.518 | 0.005 | **0.050** |
|  |  | TW (n = 6) | 5.014 | 0.025 | *0.063* | 2.000 | 0.157 | 0.225 | 0.192 | 0.061 | *0.076* | 0.264 | 0.032 | *0.064* |
|  | PAA (n = 6) | TW (n = 6) | 2.564 | 0.109 | 0.156 | 5.769 | 0.016 | **0.029** | 0.437 | 0.004 | **0.010** | 0.083 | 0.154 | 0.180 |

^1^Pairwise differences were considered significant at Q < 0.05 and are bolded, trends were considered at 0.10 < Q > 0.05 and are italicized in table

^2^Main effect and pairwise differences were determined using Kruskal-Wallis (*S.* Typhimurium: Faith’s PD P = 0.005 and Shannon’s Entropy P = 0.714; *S.* Infantis: Faith’s PD P = 0.006 and Shannon’s Entropy P = 0.001)

^3^Main effect and pairwise differences were determined using ANOSIM (*S.* Typhimurium: Jaccard P = 0.001 and Weighted Unifrac P = 0.001; *S.* Infantis: Jaccard P = 0.004 and Weighted Unifrac P = 0.001)

^4^H value: test statistic for Kruskal-Wallis

^5^R, the ANOSIM statistic, compares the mean of ranked dissimilarities between groups to the mean of ranked dissimilarities within groups
